# Comprehensive evaluation of poly(I:C) induced inflammatory response in an airway epithelial model

**DOI:** 10.14814/phy2.12334

**Published:** 2015-04-06

**Authors:** Amanda R Lever, Hyoungshin Park, Thomas J Mulhern, George R Jackson, James C Comolli, Jeffrey T Borenstein, Patrick J Hayden, Rachelle Prantil-Baun

**Affiliations:** 1 Charles Stark Draper LaboratoryCambridge, Massachusetts; 2 MatTek CorporationAshland, Massachusetts

**Keywords:** Airway epithelial cells, interleukin-8, mucus, poly(I:C)

## Abstract

Respiratory viruses invade the upper airway of the lung, triggering a potent immune response that often exacerbates preexisting conditions such as asthma and COPD. Poly(I:C) is a synthetic analog of viral dsRNA that induces the characteristic inflammatory response associated with viral infection, such as loss of epithelial integrity, and increased production of mucus and inflammatory cytokines. Here, we explore the mechanistic responses to poly(I:C) in a well-defined primary normal human bronchial epithelial (NHBE) model that recapitulates in vivo functions and responses. We developed functional and quantifiable methods to evaluate the physiology of our model in both healthy and inflamed states. Through gene and protein expression, we validated the differentiation state and population of essential cell subtypes (i.e., ciliated, goblet, club, and basal cells) as compared to the human lung. Assays for total mucus production, cytokine secretion, and barrier function were used to evaluate in vitro physiology and response to viral insult. Cells were treated apically with poly(I:C) and evaluated 48 h after induction. Results revealed a dose-dependent increase in goblet cell differentiation, as well as, an increase in mucus production relative to controls. There was also a dose-dependent increase in secretion of IL-6, IL-8, TNF-*α,* and RANTES. Epithelial barrier function, as measured by TEER, was maintained at 1501 ± 355 Ω*cm² postdifferentiation, but dropped significantly when challenged with poly(I:C). This study provides first steps toward a well-characterized model with defined functional methods for understanding dsRNA stimulated inflammatory responses in a physiologically relevant manner.

## Introduction

The epithelial barrier of the lung functions to filter particulates, pathogens, and antigens. As the first line of defense and primary target for inhaled pathogens, the upper airway elicits a dynamic immune response. The lung epithelium succeeds with a combined effort from ciliated and secretory cells (i.e., goblet and club cells) that form a pseudostratified and impenetrable barrier made up of various cell–cell junctions (Vareille et al. 2011). This physical barrier, along with effective mucociliary clearance and secreted inflammatory factors, arms the airway with distinct defense mechanisms to protect the lung (Ross et al. 2007). In major respiratory disorders, such as COPD, asthma and CF, the function of these innate mechanisms is impaired and airway infections are often worsened as a result (Crystal et al. 2008). Thus, it is essential to better understand the functional and inflammatory responses of the lung to pathogenic challenges.

Normal human bronchial epithelial cells (NHBE) isolated from human airways are an excellent model for the study of inflammation and have long been used to study airway biology and function. While other in vitro methods involved the use of airway explants, tissue fragments, and growth of cells on plastic dishes (Fulcher et al. 2005), culturing NHBE cells at an air–liquid interface (ALI) provides the appropriate culture conditions found to induce mucociliary differentiation and formation of a functional airway epithelium. This system provides an easily accessible, well-controlled and repeatable model for the study of respiratory diseases and lung inflammation (Fulcher et al. 2005). Additionally, this model facilitates the gap provided by the physiological and immunological differences found in mouse models (Blume and Davies 2013).

Well-differentiated NHBEs possess innate immune components of the upper airway and are an important model for the study of viral infection. Respiratory viruses target the epithelium of conducting airways, causing cytopathic effects and using the host to replicate and effectively shed viral particles (Matrosovich et al. 2004; Guillot et al. 2005). Toll-like receptors (TLRs) play a crucial role in recognizing pathogens and initiating a rapid immune response in the lung (Keating and Bowie 2005). Previous work confirmed that TLR3 is expressed in airway epithelial cells and is stimulated by double-stranded RNA, the intermediate produced during the viral replication process (Alexopoulou et al. 2001; Vareille et al. 2011). Once activated, TLR3 induces a potent proinflammatory stimulus for cytokine and chemokine secretion, such as, IL-8, TNF-*α*, and RANTES. Guillot et al., demonstrated that TLR3 is constitutively expressed in lung epithelial cells and that expression is upregulated by both influenza A and polyinosinic:polycytidylic acid (poly(I:C)), a synthetic analog of viral dsRNA (Guillot et al. 2005).

Poly(I:C) stimulation of airway epithelial cells has been shown to closely mimic inflammatory responses associated with viral infection. Increased cytokine production, excessive mucus secretion, loss of epithelial integrity, and impaired ciliary function can occur as a result of airway injury (Vareille et al. 2011). Poly(I:C) induced inflammation has been studied in a variety of airway epithelial cell lines, including BEAS-2B, 16HBE14o- and A549 (Guillot et al. 2005; Berube et al. 2009; Rezaee et al. 2011). Less has been established in well-characterized primary human models that more closely recapitulate in vivo physiology. Primary SAEC (small airway epithelial cells) and transfected NHBEs have been used to confirm the role of poly(I:C) induced inflammation on TLR regulation and proinflammatory cytokine secretion (Ritter et al. 2005; Melkamu et al. 2009). Additionally, well-differentiated NHBE models have been applied to study viral infection kinetics, and immune responses of host cells to different strains of influenza A infection (Gerlach et al. 2013). Gerlach et al. (2013) evaluated time-dependent inflammatory cytokine and chemokine expression after influenza infection. Cell populations were only evaluated for gene expression of cell-specific markers, without a follow-up analysis of protein. Ibricevic et al. (2006) and Matrosovich et al. (2004) focused mainly on cell population specificities to influenza and changes from infection, but did not measure barrier function, mucus production as correlated to the inflammatory response to infection. Finally, Chan et al. (2010) performed a more extensive study showing that differentiation state has an impact on the replication of influenza and innate immune responses. While cytokine and chemokine expression was monitored, TEER was not used to evaluate onset of viral infection and cell populations were not quantified (Chan et al. 2010).

While many studies have been done with primary tracheobronchial epithelial cells, most of it is focused on a specific aspect of the inflammatory response rather than recapitulation of airway function and physiology. Furthermore, the ALI system for differentiation is distinct from undifferentiated airway models in maintenance requirements and functionality, often making it difficult to compare methods and results in vitro. In response to this we have developed a set of requirements for evaluating baseline airway functions and response to inflammatory challenges in vitro. Our approach provides a framework for comparison and further advancement of biomimetic airway models. This is the first study to extensively investigate multiple dynamic responses of the airway epithelium to poly(I:C) induced inflammation in a well-characterized, primary human lung model that demonstrates innate defense mechanisms. Our goal was to examine the physical responses, as well as, the secreted inflammatory factors (i.e., IL-6, IL-8, TNF-*α*, and RANTES) that all work together to combat viral invasion. First, we confirmed that we had developed a representative lung model, with a mucociliary phenotype, by validating the presence of ciliated, basal, club, and goblet cells. We also showed that the model maintains essential physiologic functions by establishing robust and functional methods for assessing differentiation state, barrier function, mucus production, and basal levels of cytokine and uteroglobin secretion. Finally, we challenged these baseline functions with poly(I:C) and demonstrated a characteristic in vivo response to viral insult.

## Materials and Methods

### Cell culture

Cryopreserved, primary normal human bronchial epithelial (NHBE) cells from one donor were purchased from Lonza (CC2540, lot 307177, 40Y Female; Walkersville, MD). Donor had no history of smoking or preexisting lung conditions. The subjects cause of death and presence of nonlung-related diseases remains unknown. Cryopreserved cell stock was expanded in bronchial epithelial growth medium (BEGM, Lonza) at a density of 3500 cells/cm^2^. Cells were frozen down at passage 2, expanded, and seeded onto human collagen-IV (Sigma, St. Louis, MO) coated 6.5 mm Transwell inserts (Costar, Corning, NY) at a density of 3.0 × 10^5^ cells/cm². Cultures were maintained for 3 days in immersed conditions until confluency was reached. An air–liquid interface (ALI) was established by removing the apical medium and incubating with differentiation medium in the basolateral compartment only. Differentiation medium was comprised of a 50:50 mixture of LHC-basal medium (Gibco, Carlsbad, CA) and DMEM-H (Gibco) and supplemented as previously described by Fulcher et al. (2005). Cells were maintained at an air–liquid interface for up to 3 weeks, with differentiation media replenished every other day.

### Poly(I:C) induction

**Figure 1:**
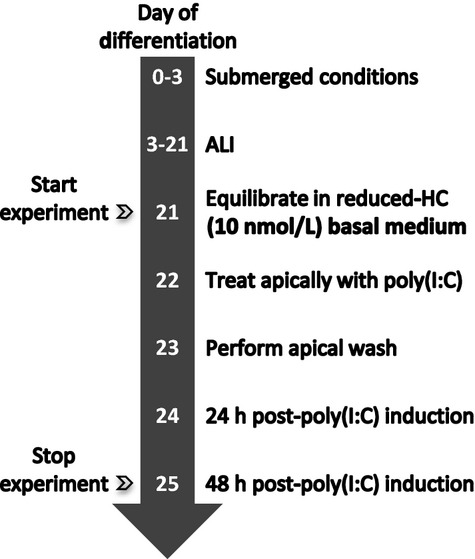
Timeline for poly(I:C) induction. NHBE cells were differentiated at an air-liquid interface (ALI) prior to start of the experiment. At day 21, cells were equilibrated in a reduced-HC differentiation medium. The next day mucus was removed and cells were treated apically with 0 *μ*g, 6 *μ*g or 12 *μ*g poly(I:C) in a 50 *μ*L volume for 24 h at 37°C. Following the incubation period, an apical wash was performed and reduced-HC basal medium was replaced. Cells were returned to the incubator and end-point analyses were run 48 h post poly(I:C) treatment.

For all poly(I:C) experiments, NHBE cells were equilibrated for 24 h in reduced-HC (10 nmol/L) differentiation media prior to induction. A high molecular weight poly(I:C) stock was prepared as indicated by manufacturer's specifications (Invivogen, San Diego, CA). Prior to addition of poly(I:C), an apical wash was performed to remove mucus. Cells were then incubated apically with 50 *μ*L PBS only (i.e., no poly(I:C) added) or 6.0 *μ*g and 12.0 *μ*g poly(I:C) in 50 *μ*L PBS for 24 h at 37°C. After the 24 h incubation period, an apical wash was performed and fresh reduced-HC media was added to the basolateral compartment. Cells were returned to the incubator for 48 h. Poly(I:C) concentrations were determined via a dose-dependent study of 3 *μ*g, 6 *μ*g and 12 *μ*g, where 3 *μ*g showed minimal changes in metrics (data not shown). For a graphical description, see Figure1.

### Immunohistochemistry and quantification

NHBE cells were validated for differentiation by immunofluorescent staining and quantification of essential cell types. Prior to staining, NHBE cells were washed with PBS and fixed with 4% Paraformaldehyde. Cells were then washed and permeabilized with 0.1% triton X-100 (Sigma) to probe for intracellular markers. Nonspecific antibody binding was blocked for 1 h with PBDT: 2% donkey serum (Jackson ImmunoResearch, West Grove, PA) and 0.01% triton X-100 in PBS. Samples were then incubated overnight at 4°C with the following primary antibodies diluted in PBDT: mouse anti-muc5AC (Thermo Scientific, Fremont, CA), mouse anti-acetylated tubulin (Sigma), and rabbit anti-CK5 (Abcam, Cambridge, MA). After incubation, cells were washed with PBS, and incubated for 1 h with corresponding fluorescent secondaries from Molecular Probes (Eugene, OR): Alexa Fluor 488 anti-mouse IgG, Alexa Fluor 546 anti-rabbit IgG or phalloidin F-actin probe. All secondary antibodies were diluted in PBDT. Cell nuclei were counterstained with Hoescht (Molecular Probes). The membrane was then excised and mounted on glass cover slides using Fluoro-gel (EMS, Hatfield, PA).

Images were obtained with an Orca-Flash4.0 LT camera (Hamamatsu, Bridgewater, NJ) on an inverted AxioObserver Z.1 microscope (Carl Zeiss, Thornwood, NY) using an EC Plan-Neofluar 40×/1.3 Oil objective (Zeiss) and 10× eyepiece. These images were analyzed with MATLAB (MathWorks, Natick, MA) and ImageJ (NIH, Bethesda, MD). A custom program was created in MATLAB to count cell nuclei and determine the total number of cells per FOV. For determination of cell-specific populations, the cell counter application in ImageJ was used to count the total number of acetylated tubulin, Muc5AC and Ck5 positive cells per FOV. Cell-specific percentages shown in Table1 are represented as the percent mean ± SEM of the total number of cells (cell nuclei) counted per FOV. Single plane confocal images were acquired with an LSM700 confocal microscope (Zeiss) equipped with a Plan-Apochomat 40×/1.3 Oil DIC M27 objective (Zeiss) operated by ZEN2012 software (Zeiss).

**Table 1:** NHBE cell population statistics at day 25 of differentiation following treatment with 0 *μ*g, 6 *μ*g, or 12 *μ*g poly(I:C). Data for each cell population were determined via image analysis and is represented as the percent mean ± SEM of the total number of cells counted per field of view (*n* = 6 replicates per treatment group, *n* = 5 FOV per replicate)

Poly(I:C) Dose (*μ*g)	% Ciliated	% Goblet	% Basal
0	20 ± 6	4 ± 1	19 ± 8
6	7 ± 5**	12 ± 1++	20 ± 3
12	4 ± 1**	22 ± 4+++	25 ± 1

^*^^*^P <* *0.01, compared with control group. ^++^*P *<* *0.01 and ^+++^*P *<* *0.001, compared with control group.

### Gene expression

All samples were collected using trizol (Qiagen, Valencia, CA) and total RNA was isolated with miRNeasy mini kit (Qiagen). RNA was quantified with a microplate reader (BioTek, Epoch, Take 3 adapter, Winooski, VT). Reverse transcription was performed from 350 ng RNA per 35*μ*L of mixture from the high-capacity reverse transcription kit (Life Technologies, Carlsbad, CA). For quantitative PCR, the following inventoried probes (Applied Biosystems, Foster City, California) were used to evaluate gene expression: Muc5AC (Hs01365616_m1), Muc5b (Hs00861588_m1), IL-6 (Hs00985639_m1), IL-8 (Hs00174103_m1), TNF alpha (Hs01113624_g1), CCL5 (Hs00171172_m1), and SCGBG1A1 (Hs00171092_m1). cDNA (3.0 *μ*L) was added to TaqMan gene expression master mix (Applied Biosystems). GAPDH (ref. seq. NM_002046.3, Applied Biosystems) served as the endogenous control.

### Mucus collection and quantification

Mucus production was measured 48 h after exposure to poly(I:C). Mucus was harvested with two apical washes of PBS (100 *μ*L each) at 37°C, a process adapted from Hill and Button (2012). Total mucus was quantified using an alcian blue colorimetric assay adapted from Hall et al. (1980). Briefly, alcian blue (Richard Allan Scientific, Kalamazoo, MI) was added to standard or samples and equilibrated for 2 h. Samples were then centrifuged at 1870 g for 30 min followed by a series of wash/spin cycles at 1870 g in a resuspension buffer consisting of 40% ethanol, 0.1 mol/L acetic acid, and 25 mmol/L MgCl_2_. Finally, the mucin pellet was dissociated with a 10% SDS (Sigma) solution in PBS and absorbance was measured with a microplate reader (Spectramax M2; Molecular Devices, Sunnyvale, CA) at 620 nm. Standard was prepared from bovine submaxillary gland mucin (Sigma) in the range of 5 *μ*g/mL to 125 *μ*g/mL.

### TEER measurements

Baseline trans-epithelial electrical resistance (TEER) was established for well-differentiated cultures as a measure of barrier function. TEER was measured using a 24-well EndOhm chamber and an EVOM2 resistance meter (WPI, Sarasota, FL). Transwell inserts were placed into the EndOhm chamber with PBS in both the apical and basal compartments. Blank Transwell inserts [with no cells] were run in conjunction with cultures to correct for background. For inflammation studies, TEER was measured 48 h post poly(I:C) induction.

### Cytokine and uteroglobin ELISAs

Inflammatory cytokines and CC10 were quantified 48 h post poly(I:C) exposure with ELISA kits from R&D Systems (Minneapolis, MN). Basal samples were collected for evaluation of IL-6, IL-8, RANTES, and CC10, and the apical wash was assayed for TNF-*α*. Assays were run according to the manufacturer's specifications.

### Statistical analyses

All statistical analyses were performed using a software package (SPSS, IBM, Armonk, NY). A one factor ANOVA was performed for all comparisons. Post hoc analyses were performed using Tukey HSD test for data with equal variance, and a Dunnett C post hoc analysis was used for data with unequal variance. All comparisons with a *P* value <0.05 were statistically significant.

## Results

### TEER and mucus production

**Figure 2:**
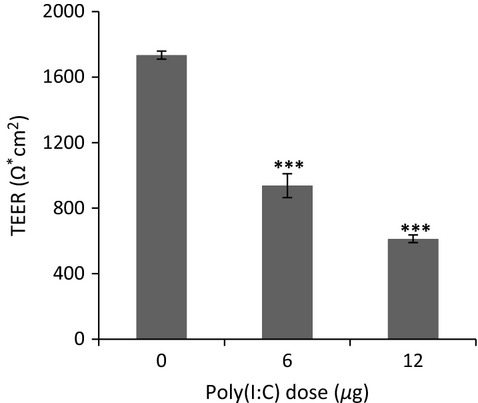
Effect of poly(I:C) induced inflammation on barrier function. TEER was measured prior to mucus removal with a 24-well Endohm chamber and an EVOM2 resistance meter purchased from WPI (Sarasota, FL). TEER data reflect a loss of barrier function 48 h after stimulation with 6 *μ*g and 12 *μ*g poly(I:C). Controls maintained baseline TEER levels. ***P <* *0.001, compared with controls. Data are expressed as the mean ± SEM (*n* = 5 biological replicates per group).

Baseline TEER was established for well-differentiated cultures as a measure of barrier function. TEER increased as the cells differentiated, reaching a value >1000 ohms*cm^2^ at over 20 days of differentiation. For poly(I:C) experiments, TEER was evaluated 48 h after induction, prior to mucosal harvest (Fig.2). A significant drop in TEER resulted after poly(I:C) exposure, where 6 *μ*g-treated NHBE cultures dropped 46% (937 ± 24 ohms*cm^2^; *P *<* *0.001) and 12 *μ*g-treated cultures decreased 65% (613 ± 24 ohms*cm^2^; *P *<* *0.001) as compared to controls (1734 ± 24 ohms*cm^2^).

**Figure 3:**
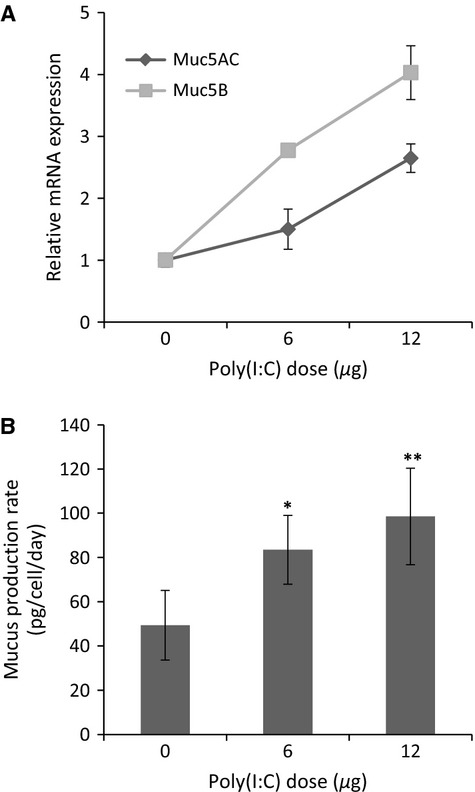
Evaluation of mucus production and mucin specific gene expression following exposure to poly(I:C). (A) mRNA expression of muc5AC (■) and muc5B (♦) was upregulated after 48 h with increasing doses of poly(I:C) (*n* = 2 biological replicates). Data are represented as the fold increase in expression relative to controls. (B) Mucus production rate was determined via an alcian blue colorimetric assay. Mucus was harvested 48 h after stimulation with poly(I:C). Results showed a significant increase in mucus production at 6 *μ*g and 12 *μ*g relative to controls. Data were normalized to the total number of cells and is represented as the mean ± SEM (*n* = 5 biological replicates per group). *P <* *0.05 and **P <* *0.01, compared with untreated controls.

Upon airlift, cells began visibly producing mucus around day 7 of differentiation, at which point, a weekly apical wash was performed to remove accumulated mucus. Mucus production was measured by alcian blue, which interacts with sulfated and carboxylated mucins. Poly(I:C) exposure increased mucus production for both 6 *μ*g (83 ± 15 pg/cell/day; *P *<* *0.05) and 12 *μ*g (100 ± 22 pg/cell/day; *P *<* *0.01) of poly(I:C) compared to controls (50 ± 16 pg/cell/day; Fig.3A). The increase in mucus production was supported by upregulation of gene expression for two specific lung mucins, Muc5AC and Muc5B (Fig.3B). Expression for both mucins increased in a dose-dependent manner at 48 h, where Muc5B (2.8 ± 0.2 for 6 *μ*g and 4.0 ± 0.2 for 12 *μ*g relative to control) expression was higher than that for Muc5AC (1.5 ± 0.4 for 6 *μ*g and 2.6 ± 0.1 for 12 *μ*g relative to control).

### Imaging

#### Quantification of cell populations and differentiation state

**Figure 4:**
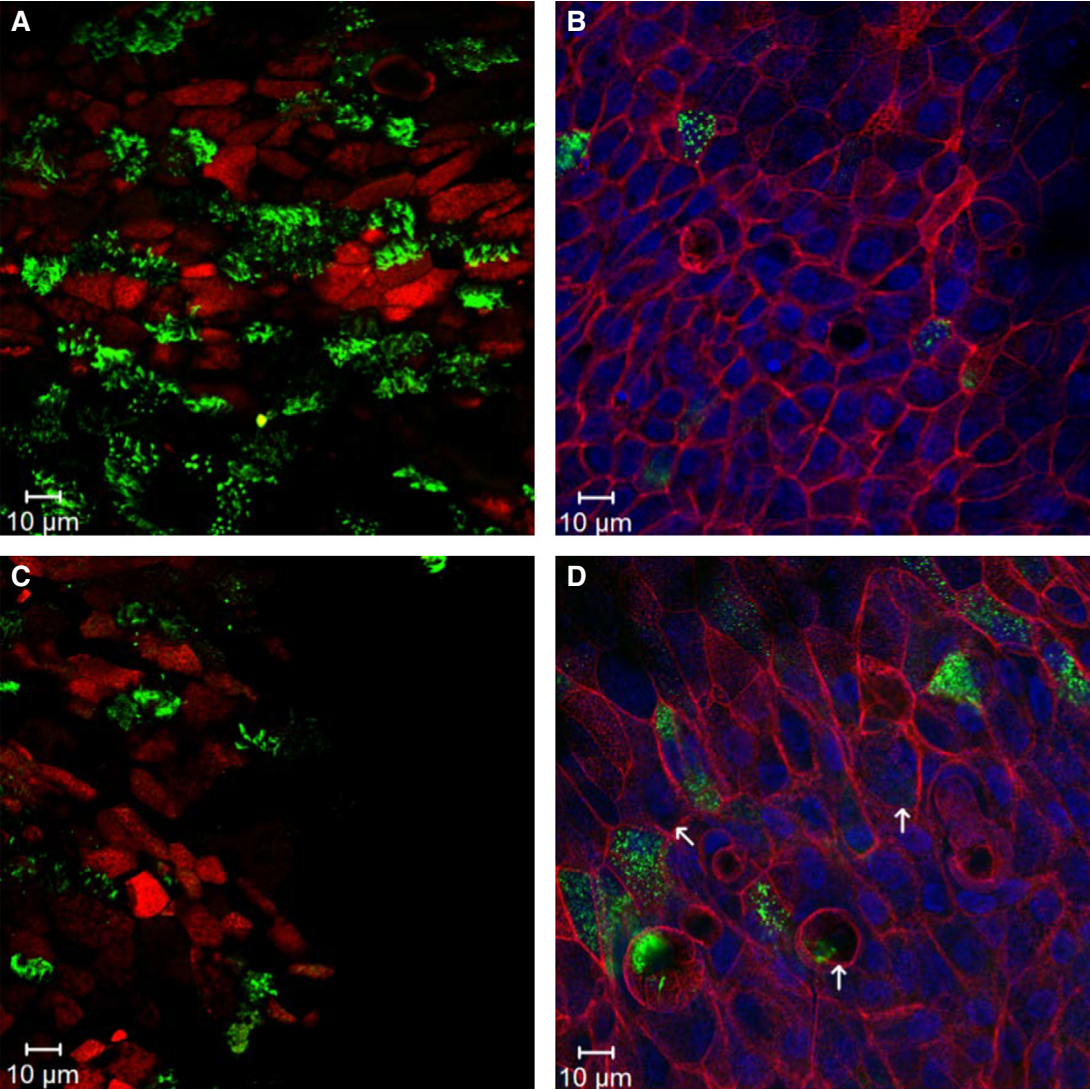
Confocal images of NHBE cells exposed to poly(I:C). Fluorescent images are shown for healthy (A-B) and inflamed (C-D) cells treated with 0 *μ*g or 6.0 *μ*g poly(I:C), respectively. Cells were fixed with 4% paraformaldehyde and probed with cell-specific antibodies 48 h after exposure. Left: Ciliated cells stained with acetylated tubulin (green) and basal cells stained with Ck5 (red). Right: Goblet cells probed with Muc5AC (green) and F-actin probed with phalloidin (red). Nuclei were counterstained with Hoescht (Blue). Arrows (D) indicate areas where cells appear elongated and rounded relative to controls. Single plane images were obtained with an LSM700 confocal microscope (Zeiss). Statistical analysis of cell populations is shown in Table1.

NHBE cells maintained at an air–liquid interface differentiated into a fully developed pseudostratified epithelium with a mucociliary phenotype. IHC analysis confirmed that the population of ciliated cells, goblet cells, and basal cells present postdifferentiation recapitulated physiologic estimates in vivo. Depicted in Fig.4A, staining with acetylated tubulin revealed populations of ciliated cells on the apical surface that accounted for 20 ± 6% of the cell population as determined by image analysis (Table1). An antibody specific to Muc5AC was used as a marker for goblet cell differentiation (Fig.4B). The population of Muc5AC positive cells was 4 ± 1% of the total number of cells present (Table1). The resident basal cell population was 22 ± 3% as indicated by cytokeratin-5 positive staining (Table1, Fig.4A). F-actin forms a tight, cobblestone morphology encompassing the Muc5AC positive cells present (Fig.4B).

#### Effect of poly(I:C) stimulation on cell subtypes

At 48 h cells were evaluated for poly(I:C) induced alteration of cell populations (Table1), as well as total number of cells. Image quantification revealed a significant loss of the total number of cells present after stimulation with 6 *μ*g of poly(I:C) (22 ± 7% decrease compared to control; *P* < 0.001) and 12 *μ*g of poly(I:C) (34 ± 5% decrease compared to control; *P* < 0.001). Poly(I:C) dramatically decreased the number of ciliated cells (Fig.4C) at 6 *μ*g and 12 *μ*g (9 ± 4%, and 3 ± 1%, respectively; *P *<* *0.01) relative to untreated controls (Table1). Additionally, analysis of muc5AC positive cells (Fig.4D) confirmed that the number of mucus producing goblet cells increased threefold after stimulation with 6 *μ*g (12 ± 1%; *P *<* *0.01, compared with control) and fivefold with 12 *μ*g (21 ± 3%; *P *<* *0.001, compared with control) of poly(I:C) (Table1). No change in the basal cell population was observed (Table1). Qualitative examination of F-actin staining conveyed an alteration in cobblestone morphology, with poly(I:C) treated cells appearing elongated and rounded relative to controls (Fig.4D). Our results indicate that stimulation with poly(I:C) alters airway epithelial cell populations and morphology, causing an upregulation in goblet cell expression and loss of ciliated cells.

### Inflammatory cytokine results

#### Gene expression analysis

**Figure 5:**
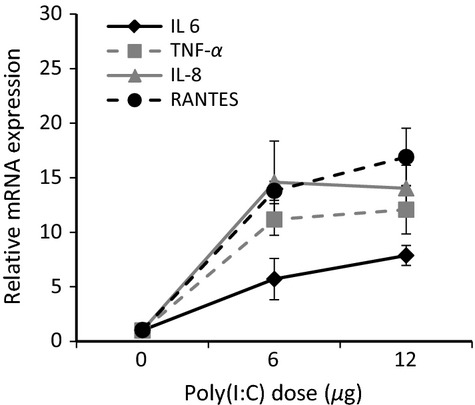
Effect of poly(I:C) stimulation on expression of inflammatory cytokines. mRNA expression was evaluated 48 h after stimulation with 0 *μ*g, 6 *μ*g or 12 *μ*g poly(I:C). Relative gene expression was upregulated for IL-6 (♦), TNF-*α* (■), IL 8 (Δ), and RANTES (•). Results are represented as the fold increase of control and were generated from two biological replicates.

Inflammatory gene expression was evaluated following the onset of poly(I:C) stimulation. Preliminary results for 24 and 48 h post poly(I:C) exposure indicated peak mRNA expression for inflammatory cytokines, TNF-*α*, IL-6, RANTES, and IL-8 at 48 h (data not shown); thus, we focused on that time point (Fig.5). Results for both 6 *μ*g and 12 *μ*g of poly(I:C) indicated robust upregulation of mRNA expression for TNF-*α* (11.2 ± 1.5, 12.1 ± 2.2, respectively), IL-6 (5.7 ± 1.9, 7.9 ± 0.9, respectively), RANTES (13.8 ± 0.9, 16.9 ± 2.6, respectively), and IL-8 (14.6 ± 3.8, 14.2 ± 2.1, respectively) relative to control. Upregulation of all cytokines peaked at the 6 *μ*g dose.

#### Cytokine analysis

**Figure 6:**
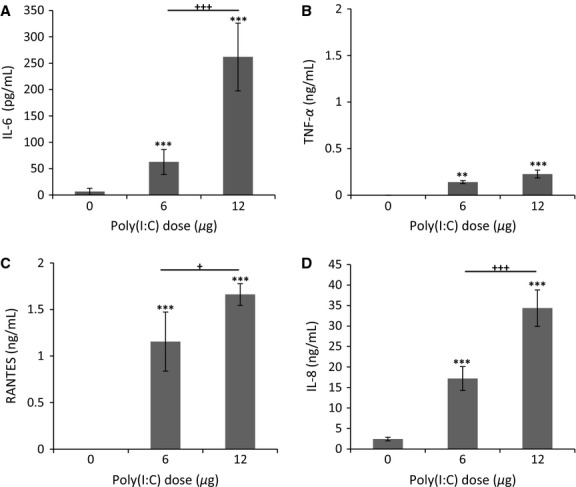
Effect of poly(I:C) induction on inflammatory cytokine and chemokine expression. Apical secretion of TNF-*α* and basal secretion of IL-6, RANTES and IL-8 was determined via ELISA 48 h after stimulation with 0 *μ*g, 6 *μ*g or 12 *μ*g poly(I:C) (*n* = 3 biological replicates). IL-6 (A) TNF-*α* (B) and RANTES (C) production increased at concentrations of 6 *μ*g and 12 *μ*g poly(I:C) relative to controls, with IL-6 and RANTES increasing in a dose-dependent manner. No protein was detected in PBS only control groups treated with 0 *μ*g poly(I:C) for both TNF-*α* and RANTES. IL-8 (D) production increased dramatically and in a dose-dependent manner compared to controls, which constituently expressed IL-8 at low levels. **P <* *0.01 and ***P <* *0.001, compared with controls. ^+^*P *<* *0.05 and ^+++^*P *<* *0.001, comparing 6 *μ*g and 12 *μ*g poly(I:C). All errors bars indicate the mean ± SEM.

Inflammatory cytokine and chemokine secretion was measured by ELISA 48 h post induction. IL-8, IL-6, TNF-*α,* and RANTES all increased in a dose-dependent manner at 6 *μ*g and 12 *μ*g poly(I:C). TNF-*α* was produced the least, with no constitutive levels measured in the controls compared to the significant increase detected with 6 *μ*g (140.6 ± 15.9 pg/mL; *P *<* *0.01) and 12 *μ*g (227.1 ± 42.5 pg/mL; *P *<* *0.001) of poly(I:C) (Fig.6B). Additionally, little IL-6 was present in controls (6.6 ± 6.3 pg/mL) compared to significant amounts produced from 6 *μ*g (62.6 ± 23.6 pg/mL) and 12 *μ*g (261.8 ± 64.2 pg/mL) of poly(I:C) (*P* < 0.01) (Fig.6A). RANTES also significantly increased with poly(I:C) exposure (1.2 ± 0.3 ng/mL for 6 *μ*g and 1.7 ± 0.1 ng/mL for 12 *μ*g; *P *<* *0.001) compared to controls, which did not produce detectable RANTES in its normal state (Fig.6C). IL-8 was constitutively expressed in controls with basal levels maintained at 2.4 ± 0.4 ng/mL (Fig.6D). The addition of 6 *μ*g and 12 *μ*g poly(I:C) led to a significant increase in IL-8 secretion (17.2 ± 2.9 ng/mL and 34.3 ± 4.4 ng/mL, respectively; *P *<* *0.001, compared with control) relative to baseline levels produced by untreated controls (Fig.5D). Overall, these results show that dsRNA is a potent stimulus for secretion of inflammatory mediators, IL-8, IL-6, TNF- *α,* and RANTES.

#### Club cells (CC10 expression)

Results for CC10 gene expression showed minimal, if any, changes in relative expression after exposure to poly(I:C) (1.9 ± 0.3 for 6 *μ*g and 1.1 ± 0.1 for 12 *μ*g relative to control). In support of these results, protein quantification for CC10 did not change for 6 *μ*g (9.1 ± 2.4 ng.mL) and 12 *μ*g (10.3 ± 1.4 ng.mL) poly(I:C) compared to controls (8.0 ± 0.7 ng.mL).

## Discussion

Utilizing a validated primary NHBE model treated with a mimic for viral dsRNA, we successfully recapitulated innate immune responses of the lung epithelium to viral infection in vitro: alteration of cell populations (Erle and Sheppard 2014), increased mucus production (Rogers 2000), damaged barrier function (Rezaee et al. 2011), and cytokine secretion (Ieki et al. 2004; Guillot et al. 2005; Melkamu et al. 2009). The results of this study indicate that our in vitro lung model, when inflamed with poly(I:C), imitates in vivo responses of the human lung to viral infection. This well-defined approach allows us to better understand the onset of inflammatory response to various cues (e.g., virus, allergens, bacteria, etc.) and how it relates to disease states such as asthma and COPD. As a result, we were able to correlate changes in cytokine secretion and signaling pathways to in vivo physiologic metrics, which may help to further identify new pathways, targets, or therapeutics for obstructive airway disease.

Airway epithelial cells express TLR3, a pattern recognition receptor that when activated by viral dsRNA initiates a rapid inflammatory response (Greene and McElvaney 2005; Ritter et al. 2005; Rezaee et al. 2011). This includes increased secretion and expression of inflammatory cytokines and chemokines. In this study, we demonstrate that poly(I:C), a TLR3 ligand, induces upregulation of TNF-*α*, IL-8, RANTES, and IL-6 (Fig.6). We chose to assay these inflammatory mediators because they are known to contribute to the onset of airway inflammation (Vareille et al. 2011). Respiratory viruses induce RANTES and IL-8 production, causing an amplified inflammatory response known to exacerbate conditions of COPD and asthma (Ieki et al. 2004; Berube et al. 2009). IL-8 and RANTES are from different families of chemokines that recruit neutrophils (Qiu et al. 2007; Vareille et al. 2011) and eosinophils (Berube et al. 2009), respectively. Our results revealed a dramatic increase in mRNA expression and secretion of both IL-8 and RANTES following poly(I:C) stimulation (Figs5 and Fig.6C–D). This data are in agreement with a study on TLR3 activation by poly(I:C) in immortalized Beas-2b bronchial epithelial cells. Berube and colleagues (Berube et al. 2009) reported a strong upregulation of both RANTES and IL-8 following stimulation with 10 *μ*g/mL poly(I:C) for 24 h. Although this inclination agrees with our results, we noted a larger increase in protein expression of IL-8 and RANTES after poly(I:C) treatment, which could be due to our longer incubation period or the use of a well-differentiated model as opposed to a less physiologic cell line.

Secretion of acute phase inflammatory cytokines, such as TNF-*α*, is another essential component of the antiviral-mediated response. TNF-*α* is produced by airway epithelial cells upon infection and induces recruitment of macrophages, as well as enhanced secretion of mucus and lung permeability (Krunkosky et al. 2000; Hardyman et al. 2013). TNF-*α* is also thought to stimulate IL-6 secretion, which acts to stimulate cellular defense mechanisms (Cromwell et al. 1992; Krunkosky et al. 2000). Our analysis revealed a 10-fold increase in TNF-*α* mRNA expression at both poly(I:C) concentrations and a dose-dependent increase in IL-6 mRNA expression of up to sevenfold relative to untreated controls (Fig.5). While IL-6 secretion increased dramatically, only a moderate increase in TNF-*α* secretion was observed at the highest dose (Fig.6A–B). In a study by Melkamu et al. (2009), well-differentiated NHBE cells (hTERT derived) were inflamed with 25 *μ*g/mL poly(I:C) and basal secretion of cytokines was evaluated at 24 h. They reported a much higher increase in IL-6 and RANTES than TNF-*α*, compared to untreated controls. A 50-fold increase in IL-6 mRNA expression was also reported (Melkamu et al. 2009). Overall, the general upregulation of cytokine expression we observed agreed with this previous study. Additionally, increased levels of these inflammatory cytokines was observed in serum and nasal lavage fluid collected from patients infected with influenza A (Hayden et al. 1998).

Along with cytokine production, phenotypic alterations are another strong indicator of an inflammatory response in vivo. Viral respiratory infections, in particular, can alter cell phenotypes and population densities in the human lung (Ibricevic et al. 2006). In our in vitro study, image analysis confirmed the alteration of goblet, ciliated and basal cells present after exposure to poly(I:C) (Table1). Healthy controls were comparable to in vivo estimates of a normal upper airway, which is comprised of 30–50% ciliated cells, and ∼1–13% mucus producing or goblet cells (Mercer et al. 1994; Boers et al. 1999). Poly(I:C) treated cultures revealed significantly decreased ciliated cell populations and increased goblet cell populations (Table1). Maintenance of these cell populations is critical for mucociliary transport, a coordinated process in which the mucus layer rests on beating cilia, effectively trapping and clearing pathogens from the airway (Hill and Button 2012). Disruption of this mechanism hinders the lungs ability to fight infection, allowing viruses and other pathogens to thrive.

The population of Ck5 positive cells is also significant as basal cells are essential for cell turnover, differentiation, and epithelial repair (Crystal et al. 2008). Our Ck5 analysis of healthy controls was representative of the 30% basal cells estimated in a healthy upper airway in vivo (Mercer et al. 1994; Rock et al. 2010). We saw no significant change in the number of Ck5 positive cells after poly(I:C) induced injury (Table1). This may be limited by a single time point of evaluation (Musah et al. 2012), as well as the use of only one marker for identification of basal epithelial stem cells and repair of epithelial injury (Rock et al. 2010, 2011).

Club cells, a secretory cell population, also behave like basal stem cells by providing another level of plasticity within the epithelium for injury repair and protection (Broeckaert and Bernard 2000). In vivo, CC10 has an important antiinflammatory role with xenobiotic metabolizing capabilities (Broeckaert and Bernard 2000), and has been linked to inflammatory cytokines, including TNF-*α*, which was shown to stimulate CC10 production in undifferentiated NHBTE cells at a concentration of 20 ng/mL (Yao et al. 1998). We evaluated the effect of poly(I:C) on CC10 secretion. In our analysis, we observed no change in CC10 production with a poly(I:C) induced secretion of ∼0.2 ng/mL TNF-*α* (Fig.6B). Regardless, the role of CC10 in this pathway requires additional investigation.

Injury to the epithelial barrier of the airway can cause loss of epithelial integrity and airway homeostasis (Vareille et al. 2011). Respiratory viruses are known to induce barrier disruption and dissociation of tight junction proteins, ZO-1 and occludin, causing increased epithelial permeability (Comstock et al. 2011). Our results demonstrate that exposure of the airway to poly(I:C) leads to significant alterations in barrier function. TEER evaluation of barrier function revealed a dose-dependent drop of 50 and 65%, at concentrations of 6.0 *μ*g and 12.0 *μ*g, respectively, relative to controls (Fig.2). These findings are consistent with a previous study that treated differentiated NHBE cells with 5 *μ*g/mL poly(I:C) and reported a ∼60% drop in TEER at 48 h, relative to untreated cells (Rezaee et al. 2011). The study also compared these values to poly(I:C) treated 16HBE14o- cells and found a difference in TEER kinetics, with loss of barrier function occurring more slowly in NHBE cells (Rezaee et al. 2011).

Viral dsRNA can also cause cell death, morphology changes and loss of ciliated cells (Ibricevic et al. 2006; Vareille et al. 2011), which we believe also contributed to loss of barrier function. After poly(I:C) induction, image analysis revealed a dramatic decrease in the population of ciliated cells relative to untreated populations (Table1). There was also a loss in the total number of cells at the highest dose of poly(I:C) pointing to cell death as a probable cause for loss of cilia. Another explanation may be that ciliated cells participate in epithelial repair after acute injury through transdifferentiation (Park et al. 2006). Qualitative observation of F-actin staining indicated that poly(I:C) treatment altered cobblestone morphology relative to untreated controls (Figs4B and D). Modification to the tight cobblestone morphology and loss of ciliated cells can be attributed to epithelial injury, as well as loss of barrier function via insult with dsRNA.

As a result of poly(I:C) treatment, we noted a 40–50% increase in mucus production, as well as, a marked increased in goblet cells and mucin gene expression (Fig.3A–B; Table1). These results support findings that attribute mucus hypersecretion in airway disease to an upregulation of mucin genes, along with goblet cell hyperplasia (Chen et al. 2003; Rose and Voynow 2006). The increase in total mucus, after 48 h of poly(I:C) treatment, correlated with a three- to fourfold increase of Muc5B mRNA expression and a 1.5- to 2.5-fold upregulation of Muc5AC mRNA (Fig.3A). Muc5AC is primarily produced by goblet cells at the surface of the epithelium and is thought to respond to acute insults of the upper airway. Muc5B on the other hand, is produced mainly in the submucosal glands as a result of chronic insults and disease (Thornton et al. 2008). Several factors are involved in stimulating mucin expression and secretion, including influenza and inflammatory cytokines (Rose and Voynow 2006). Thus, we conclude that the increase in mucin gene expression and mucus secretion in our NHBE model is due to stimulation with poly(I:C) and the resulting increase of inflammatory cytokines, TNF-*α*, IL-8, and IL-6 (Fig.6). In support of this, previous work has shown that TNF-*α* and IL-8 increase Muc5AC expression in epithelial cells by regulating at the posttranscriptional level and increasing mRNA stability (Bautista et al. 2009). Additionally, IL-6 has been reported to increase Muc5AC and Muc5B steady-state expression in NHBE cells (Chen et al. 2003). Along with the upregulation in mucin mRNA expression, our results revealed a substantial increase in the number of mucus producing cells (Table1). Cytokine induced elevation of Muc5AC positive cells has also been reported in NHBE cells treated with IL-17A and IL-1*β*, common cytokines that play a role in chronic airway disease (Fujisawa et al. 2009).

While our study provides a sufficient platform to study inflammatory damage to the upper airway, there are some limitations. The results present only one donor. It is important to understand how donor to donor variability may alter the approach and results for poly(I:C) treatment. Some patients with interferon (an antiviral agent released by the epithelium during infection) deficiencies may be more susceptible to infection (Wark et al. 2005). It is also difficult to obtain the state of the cadaveric donor airway, which may be an important factor in the poly(I:C) response (Ghosh et al. 2013). An additional limitation is that aqueous delivery of poly(I:C) may not be comparable to aerosolized in vivo delivery of a virus. Finally, this model lacks an immune component which has been shown to enhance inflammatory responses in vitro, as well as provide a more realistic model of inflammation (Blume and Davies 2013).

In conclusion, we present a well-defined approach for understanding virally induced inflammation of a physiological lung epithelial model with functional and quantifiable metrics that recapitulate in vivo responses. Stimulation with a sufficient amount of poly(I:C) altered baseline cell populations, mucus production and barrier function, in conjunction with enhanced secretion of inflammatory cytokines and chemokines. This study provides a basis for a coculture model with immune cells added to capture the cellular interplay that is crucial to pathogenic or allergenic inflammatory responses. The promise of these primary cultures is to provide an isolated, controlled, and physiological platform that advances research of the airway epithelium in health and disease states. A repeatable model and approach will progress the understanding of chronic airway disease, as well as development of alternative therapeutic modalities.

## Conflict of Interest

None declared.
